# Heredity with a Study of the Statistics of the New York State Hospitals1Read before the Section on Medicine, Buffalo Academy of Medicine, May 13, 1902.

**Published:** 1902-07

**Authors:** William C. Krauss

**Affiliations:** Buffalo, N. Y.; Consulting Neurologist, Buffalo General Hospital, Providence Retreat; Attending Neurologist, Erie County, German, German-Deaconess, Emergency and Woman’s Hospitals, Buffalo Eye and Ear Infirmary; Fellow of the American Neurological Association


### ABSTRACT.

### Heredity with a Study of the Statistics of the New York State Hospitals.[1]

By WILLIAM C. KRAUSS, M. D., Buffalo, N. Y.

Consulting Neurologist, Buffalo General Hospital, Providence Retreat; Attending Neurologist, Erie County, German, German-Deaconess, Emergency and Woman's Hospitals, Buffalo Eye and Ear Infirmary; Fellow of the American Neurological Association.

HEREDITY has been defined as that peculiar property of an organism which transmits to its offspring the characteristics of its progenitors. If those characteristics are ones of grace, beauty and strength, the offspring will inherit the corresponding qualities of the parent; if, on the other hand, defect and infirmity are the characteristics of the sire, then these qualities will reappear in the young, often with renewed impetus and reinforcement. Moreover, while it is an easy matter for the higher and nobler attributes to become through custom and environment deflected and deteriorated it is almost an impossibility for the baser and decrepit qualities and conditions to become regenerated and rehabilitated.

These laws hold good not only in the human family but in the vegetable and animal worlds as well; they follow exactly the same course and terminate at the same place. Out of propagated weakness there cannot come strength; out of defects there cannot come perfection. To me heredity is nothing more than a mirror reflecting from one generation to another the grace, beauty and strength, or else the coarse, ugly, defective features of the one standing before it.

Heredity may also be compared to a composite photograph. If the facial lines of the individuals selected are regular, the facial

1. Read before the Section on Medicine, Buffalo Academy of Medicine, May 13, 1902.

angles of nearly equal degree, and the component parts symmetrical, the photograph will be clear, sharp and a good reproduction of the individual features.

If there exists asymmetry of the two facial halves, if the component parts are deformed, irregular and the facial angles of the individuals are of varying degrees, a composite photograph will be obtained that is blurred, distorted and defective. So also with a child of faulty parentage or grandparentage,—it represents the ensemble of the vices and defects of successive generations, unless arrested or attenuated by environment. As Kiernan has well said, Heredity is a prophecy of what may be, not necessarily a destiny which must be.

The diagnostic value of a hereditary tendency to insanity depends largely on its degree. Thus the insanity of one parent would indicate a less degree of predisposition than that of one parent and an uncle, or still less than that of a parent and a grandparent, or of both parents. Again, the insanity of a parent and a grandparent with an uncle or an aunt in the same line may be held to indicate a stronger predisposition than even the insanity of both parents.

The significance of the insanity of parents will depend to a large extent upon the period of its onset. The insanity of a parent occurring after the birth of a child, if it arose from a cause adequate to excite it without previous predisposition, would be held, of course, as of no value in the formation of a hereditary tendency.

The insanity of relatives further removed than parents, uncles and aunts, brothers and sisters and first cousins, is not worth anything except in corroboration of nearer and weightier facts. But the influence of other related diseases to insanity occurring in those near akin, such as eccentricity, alcoholism, epilepsy, hysteria, hypochondriasis, vicious or criminal tendencies, and the like, may be of great import.

Perhaps nowhere is this doctrine of heredity more potent than in psychiatry; and in the statistics gathered and published by the different hospital systems, lunacy commissions and census reports, one can gain some idea of its strength and solidity.

The New York State Lunacy Commission, with the large and well conducted hospitals under its supervision, is able to furnish some figures on the frequency of heredity which must carry some weight and conviction even to those who are inclined to reject the doctrine or those who believe that its importance has been overestimated. The tables quoted are taken from the last annual report of the New York State Lunacy Commission, composed of Dr. Frederick Peterson, chairman, W. L. Parkhurst, Daniel N. Lockwood and T. E. McGarr, secretary.

RECAPITULATION.

*Showing percentage of heredity of the twelve State Hospitals for the year 1899–1900 and since October 1, 1888.*

| INSTITUTIONS. | 1899–1900. | | | Since October 1, 1888. | | |
|---|---|---|---|---|---|---|
| | Heredity of whole number. | Exclusive of unascertained. | No heredity exclusive of unascertained. | Heredity of whole number. | Exclusive of unascertained. | No heredity exclusive of unascertained. |
| Utica S. H. . . . | 26.5 | 30. | 70. | 29.6 | 51.4 | 48.5 |
| Willard S. H. . . . . . | 33.8 | 41.1 | 58.8 | 28.6 | 46.8 | 53.5 |
| Hudson River S. H. . . | 34.7 | 44.5 | 55.4 | 27.5 | 53.3 | 46.6 |
| Middletown S. H. . . | 26.4 | 28.2 | 71.7 | 29.4 | 31.6 | 68.7 |
| Buffalo S. H. . . . . . | 24.7 | 30.9 | 69. | 21.1 | 31.6 | 68.3 |
| Binghamton S. H. . . . | 36.2 | 40.1 | 59.8 | 32.9 | 43.4 | 56.5 |
| St. Lawrence S. H . . | 35.8 | 52.1 | 47.8 | 33.8 | 50.7 | 49.2 |
| Rochester S. H. . . . . | 31.6 | 34.1 | 65.8 | 27.4 | 39. | 60.9 |
| Long Island S. H. . . . | 17.5 | 33.9 | 69. | 15.7 | 32.6 | 67.3 |
| Manhattan S. H. East | 13.4 | 14.2 | 82.7 | 13.1 | 18.1 | 75.9 |
| Manhattan S. H. West | 11.7 | 14.1 | 85.7 | 14. | 18.6 | 81.5 |
| Gowanda S. H. . . . | 28.5 | 36. | 63.9 | 31.2 | 42.9 | 57. |
| Matteawan S. H. . . | 15.4 | 76.4 | 23.2 | 15. | 56. | 43.9 |

Percentage showing heredity, 1899-1900, 25.8; since 1888, 24.5.

Percentage exclusive of unascertained, 1899-1900, 36.6; since 1888, 39.7.

Percentage showing no heredity exclusive of unascertained, 1899-1900, 63.3; since 1888, 59.8.

Total number of cases admitted, 1899-1900, 6,361; since 1888, 61,257.

Total number of hereditary cases, 1899-1900, 1,202; since 1888, 14,526.

The hospital showing the highest percentage of heredity of the whole number for the year 1895-1896 was the Matteawan Hospital, with a percentage of 57.1; for the year 1898-1899, the St. Lawrence Hospital with a percentage of 41.6; for the year 1899-1900, the Binghamton Hospital with a percentage of 36.2. And since 1888, the St. Lawrence Hospital with a percentage of 33.8.

Exclusive of the unascertained cases the St. Lawrence Hospital showed the highest percentage for the year 1898-1899, with 56.6; for the year 1899-1900, the Matteawan Hospital with a percentage of 76.4; and since 1888, the Matteawan Hospital with a percentage of 76.4.

The lowest percentage of heredity for the year 1895-1896 was shown by the Manhattan State Hospital with a percentage of 17.3; 1898-1899, the Matteawan Hospital with a percentage of 15.3; 1899-1900, the Manhattan Hospital, West, with a percentage of 11.7; and since 1888, the Manhattan Hospital, East, with a percentage of 13.1.

Exclusive of the unascertained cases the Manhattan Hospital showed the lowest percentage for the year 1898-1899, with a percentage of 16.6; the Manhattan Hospital, West, for the year 1899--1900, with a percentage of 14.1; and since 1888, the Manhattan Hospital, East, with a percentage of 18.1.

These percentages vary of course from year to year and the percentage of the whole number since 1888, as 39.7, is perhaps as correct an index of the true percentage as it is possible to determine.

It is a noteworthy fact that the reports of the New York State Hospital show that *maternal* transmission is increasing rapidly over *paternal* transmission, as shown by comparison of the figures given for the years 1895-1896 and 1899-1900.

Turning now to nervous diseases proper, we find heredity just as strongly represented in the various neuroses as was found for the psychoses,—there is transmitted in the organism certain diatheses which favor certain diseases, such as Huntington's chorea, Friedreich's disease, running through successive generations. These diseases are termed hereditary, familial, embryonic, and this succession is what is meant by the term direct heredity or organic heredity. The severity of the heritage depends very largely upon the number of members and branches affected. Here again, as in the study of psychotic heredity, we find that maternal transmissibility far exceeds the paternal.

Indirect heredity is heredity by transformation from other neuropsychic diseases and is more common but of less consequence than direct heredity. Given a neuron feebly endowed with enduring qualities, it is not improbable that any condition capable of reducing the general health may act with unusual virulence upon it. The result is a neuropathic disposition, or a nervous organisation with a tendency to yield readily to undue strains and unusual influences, though of themselves of no material importance. There is propagated from parent to offspring certain diatheses which favor certain neuropathic equivalents. Thus epilepsy, melancholia or inebriety, may favor the production of hysteria, chorea or neurasthenia, while in the succeeding generations, the transformation of the neuroses and toxic diatheses in propagation result often in imbecility. Thus the children of hysteric, epileptic, hypochondriac and syphilitic or alcoholic parents are liable to be imbecile. Phthisical parents also frequently beget imbecile children.

In the progressive degeneracy which leads to the extinction of families, imbecility is the next to the final stage, which ends with idiotic incapacity of reproduction. (Kellogg.)

There is thus nutured a family tree whose branches become heavily laden with neuropathic fruit, yielding and bending to the slightest zephyr until through sterility it becomes barren and lifeless and falls by the wayside in the struggle for existence.

The diseases of the nervous system are arranged alphabetically, according to their hereditary tendency, and also those which authorities are agreed do not belong to this category. The hereditary neuroses are divided into those of direct and indirect transmissibility.

Abscess, cerebral; no heredity. Acromegaly; no heredity. Adiposis dolorosa; indirect heredity. Amyotrophic lateral sclerosis; indirect heredity occasionally and direct. Anemia, cerebral; no heredity. Aneurism, intracranial; direct heredity occasionally. Angioneurotic edema; direct heredity. Apoplexy, cerebral (cerebral arterial disease); direct heredity very slight. Astasia abasia; direct heredity. Bell's palsy; no heredity. Brown-Sequard's paralysis; no heredity. Bulbar paralysis, asthenic; no heredity. Bulbar paralysis, progressive; indirect heredity. Caisson disease; no heredity. Chorea, electrical (Dubini's disease); no heredity. Chorea, Huntington's; direct heredity. Chorea, Sydenham's; indirect heredity. Coprolalia; indirect heredity. Cretinism, endemic; direct heredity. Eclampsia infantum; no heredity. Embolism, cerebral; no heredity. Encephalitis, acute hemorrhagic; no heredity. Epilepsy; direct heredity, 33 per cent. paternal. Erythromelalgia; no heredity. Exophthalmic goitre; indirect and direct heredity. Hematomyelia; no heredity. Hematorrhachis; no heredity. Hemiatrophia facialis; direct heredity. Hereditary amaurotic idiocy; direct heredity. Hereditary ataxic paraplegia; direct heredity. Hereditary cerebellar ataxia; direct heredity. Hereditary cerebral diplegia; direct heredity. Hereditary hemiplegia; direct heredity. Hereditary spastic paraplegia; direct heredity. Herpes zoster; no heredity. Hydrocephalus, chronic; indirect heredity. Hyperemia, cerebral; no heredity. Hyperostosis cranii; no heredity. Hypochondriasis; direct and-indirect heredity. Hysteria; direct heredity 75 per cent. maternal. Landry's paralysis; no heredity. Laryngismus stridulus; indirect heredity. Leptomeningitis, acute; no heredity. Leptomeningitis, chronic; no heredity. Little's disease, congenital; direct heredity. Locomotor ataxia; indirect heredity. Menière's disease; no heredity. Meningitis, alcoholic, serous; no heredity. Meningitis, epidemic cerebrospinal; no heredity. Meningitis, tuberculous; direct heredity. Migraine; direct heredity. Morvan's disease; no heredity. Muscular atrophy, arthritic; no heredity. Muscular atrophy (Charcot-Tooth); direct heredity. Muscular atrophy (Duchenne-Aran); indirect heredity. Muscular atrophy, occupation; no heredity. Muscular dystrophy, progressive (Landouzy-Déjerine and Erb types); direct heredity. Muscular hypertrophy, pseudo; direct heredity. Myelitis, acute, transverse; indirect heredity. Myelitis, chronic; no heredity. Myxedema; no heredity. Neuralgias; indirect heredity. Neuritis; indirect heredity, tendency to alcoholism. Neuromata, true, multiple and plexiform; direct heredity. Occupation neurosis; indirect heredity. Ophthalmoplegia, progressive; no heredity. Pachymeningitis cervicalis hypertrophica; no heredity. Pachymeningitis hemorrhagica interna; no heredity. Paralysis agitans; direct heredity (rare). Paramyoclonus, multiplex; indirect heredity. Paresis; indirect heredity. Poliomyelitis acuta adultorum; no heredity. Poliomyelitis acute anterior; 1–2 per cent. direct heredity. Rabies; no heredity. Raynaud's disease; indirect heredity. Saltatoric spasm; indirect heredity. Scleroderma; indirect heredity. Sclerosis, combined of anemia; no heredity. Sclerosis, multiple; indirect and direct heredity. Spasm, facial; indirect heredity. Spasm, habit; indirect heredity. Spina bifida; direct heredity. Syphilis, cerebral; indirect and direct heredity. Syringomyelia; no heredity. Tetanus; no heredity. Tetany; no heredity. Thomsen's disease; direct heredity. Thrombosis, cerebral; no heredity. Torticollis; direct and indirect heredity. Tumors, cerebral; no heredity. Tumors, spinal, no heredity.

**Direct Heredity.**

- Amyotrophic lateral sclerosis.
- Aneurism, intracranial.
- Angioneurotic edema.
- Apoplexy, cerebral.
- Astasia abasia.
- Chorea, Huntington's.
- Cretinism.
- Epilepsy.
- Exophthalmic goitre.
- Hemiatrophia facialis.
- Hereditary amaurotic idiocy.
- Hereditary ataxic paraplegia.
- Hereditary cerebellar ataxia.
- Hereditary cerebral diplegia.
- Hereditary hemiplegia.
- Hereditary spastic paraplegia.
- Hypochondriasis.
- Hysteria.
- Little's disease.
- Meningitis, tuberculous.
- Migraine.
- Muscular atrophy (Charcot-Tooth.)
- Muscular dystrophy (Landouzy-Déjerine and Erb types.)
- Muscular hypertrophy, pseudo.
- Neuromata.
- Paralysis, agitans.
- Poliomyelitis, acute anterior.
- Sclerosis, multiple.
- Spina bifida.
- Thomsen's disease.
- Torticollis.

**Indirect Heredity.**

- Adiposis dolorosa.
- Amyotrophic lateral sclerosis.
- Bulbar paralysis, progressive.
- Chorea, Sydenham's.
- Coprolalia.
- Exophthalmic goitre.
- Hydrocephalus, chronic.
- Hypochondriasis.
- Hysteria.
- Laryngismus stridulus.
- Locomotor ataxia.
- Muscular atrophy (Duchenne-Aran.)
- Myelitis, acute transverse.
- Neuralgias.
- Neurasthenia.
- Neuritis
- Occupation neuroses.
- Paramyoclonus, multiplex.
- Paresis.
- Raynaud's disease.
- Saltatoric spasm.
- Scleroderma.
- Sclerosis, multiple.
- Spasm, facial.
- Spasm, habit.
- Syphilis, cerebral.
- Torticollis.

No Heredity:

Abscess, cerebral.
Acromegaly.
Anemia, cerebral.
Bell's palsy.
Brown-Sequard's paralysis.
Bulbar paralysis, asthenic.
Caisson disease.
Chorea, electrical; Dubini's disease.
Eclampsia infantum.
Embolism, cerebral.
Encephalitis, acute hemorrhagic.
Erythromelalgia.
Hematomyelia.
Hematorrhachis.
Herpes Zoster.
Hyperemia, cerebral.
Hyperostosis cranii.
Landry's paralysis.
Leptomeningitis acuta.
Leptomeningitis chronica.
Menière's disease.
Meningitis alcoholic (serous).
Meningitis, epidemic cerebrospinal.
Morvan's disease.
Muscular atrophy, arthritic.
Muscular atrophy, occupation.
Myelitis, chronic.
Myxedema.
Ophthalmoplegia, progressive.
Pachymeningitis cervicali hypertrophica.
Pachymeningitis hemorrhagica interna.
Poliomyelitis acuta adultorum.
Rabies.
Sclerosis, combined of anemia.
Syringomyelia.
Tetanus.
Tetany.
Thrombosis, cerebral.
Tumors, cerebral.
Tumors, spinal.

---

